# Role of CD155/TIGIT in Digestive Cancers: Promising Cancer Target for Immunotherapy

**DOI:** 10.3389/fonc.2022.844260

**Published:** 2022-03-30

**Authors:** Daijun Wang, Yanmei Gu, Xin Yan, Chengdong Huo, Guan Wang, Yang Zhao, Muzhou Teng, Yumin Li

**Affiliations:** ^1^ The Second Clinical Medical College of Lanzhou University, Lanzhou University, Lanzhou, China; ^2^ Key Laboratory of Digestive System Tumors of Gansu Province, Second Hospital of Lanzhou University, Lanzhou, China

**Keywords:** TIGIT, CD155, T cells, NK cells, gastric cancer, liver cancer, pancreatic cancer, colorectal cancer

## Abstract

The tumor microenvironment restricts the function and survival of various immune cells by up-regulating inhibitory immune checkpoints, and participates in the immune escape of tumors. The development of immunotherapies targeting immune checkpoints, such as programmed cell death receptor 1 antibody and anti-cytotoxic T lymphocyte-associated antigen 4 antibody, has provided many options for cancer treatment. The efficacy of other immune checkpoint inhibitors is also under development and research. Among them, T cell immunoreceptor with Ig and ITIM domains (TIGIT) has shown excellent clinical application prospects. Correspondingly, poliovirus receptor (PVR, CD155), one of the main ligands of TIGIT, is mainly expressed in various human malignant tumors and myeloid cells. CD155 interacts with TIGIT on natural killer cells and T cells, mediating inhibitory immunomodulatory regulation. This study summarized the mechanism of CD155/TIGIT in regulating immune cells and its role in the occurrence and development of digestive system tumors, aiming to provide a new perspective for immunotherapy of digestive cancers.

## Introduction

Immune checkpoints are stimulatory and inhibitory receptors of the immune system. The inhibitory immune checkpoints are involved in regulating immune activation, preventing excessive activation of immune responses, and maintaining immune tolerance. Tumor cells can achieve immune escape by hijacking inhibitory immune checkpoints, such as programmed cell death-1 protein/ligand (PD-1/PD-L1), cytotoxic T lymphocyte antigen 4 (CTLA4), T cell immunoglobulin and mucin domain-containing protein 3 (TIM-3), which regulates the function and survival of immune cells and facilitates immune suppressive microenvironment (1, 2). At present, immune checkpoint inhibitors (ICIs) have been developed for clinical application, which block the interaction between tumor cells expressing inhibitory immune checkpoints and immune cells and reinvigorate anti-tumor immune responses. Nivolumab and pembrolizumab, ICIs that target PD-1, showed promising anti-tumor activity in patients with non-small cell lung carcinoma, with an objective response rate of 56% and 45.2%, respectively (3, 4). In patients with advanced cutaneous squamous-cell carcinoma, the response to PD-1 inhibitors was 47% (5). Melanoma patients treated with CTLA-4 inhibitors exhibited improved survival (6). However, multiple clinical trials revealed that various types of tumors, such as pancreatic ductal adenocarcinoma (7), esophageal adenocarcinoma (8) and prostate cancer (9), exhibited poor responses to ICIs. Furthermore, the clinical benefits of ICIs are also accompanied by severe immune toxicity, including cardiotoxicity (10), pneumonia (11), hepatitis,colitis (12), pancreatitis (13) and endocrine dysfunction (14). The excessive immune cell responses caused by ICIs, including the activation of T cells and macrophages, produce many proinflammatory cytokines and induce a life-threatening inflammatory response (15, 16). Therefore, it might be promising to develop new immune checkpoints targets to optimize treatment regimens and reduce cytotoxicity.

Natural killer (NK) cells and T cells represent the central part of the immune system (17, 18), and inhibitory immune checkpoints expressed on their surfaces are gradually being explored. T cell immunoreceptor with Ig and ITIM domains (TIGIT) is considered to be an inhibitory receptor in activated T cells, NK cells, and Treg cells (18–20), and has become a promising target for immunotherapy. Poliovirus receptor (PVR, CD155) is the ligand with the highest binding affinity to TIGIT, which plays a critical role in cell adhesion, migration, proliferation and regulation of immune responses (21). The interaction between TIGIT and CD155 can mediate the functional exhaustion and hyporesponsiveness of lymphocytes, and induce the immune escape of tumor cells. This emerging immune checkpoint is expected to become a novel target for tumor immunotherapy (22–24). This review summarizes the biological function and mechanism of TIGIT/CD155 in immune cells and the latest research progress in digestive system tumors.

## Expression and Function of CD155/TIGIT

**Figure 1:**
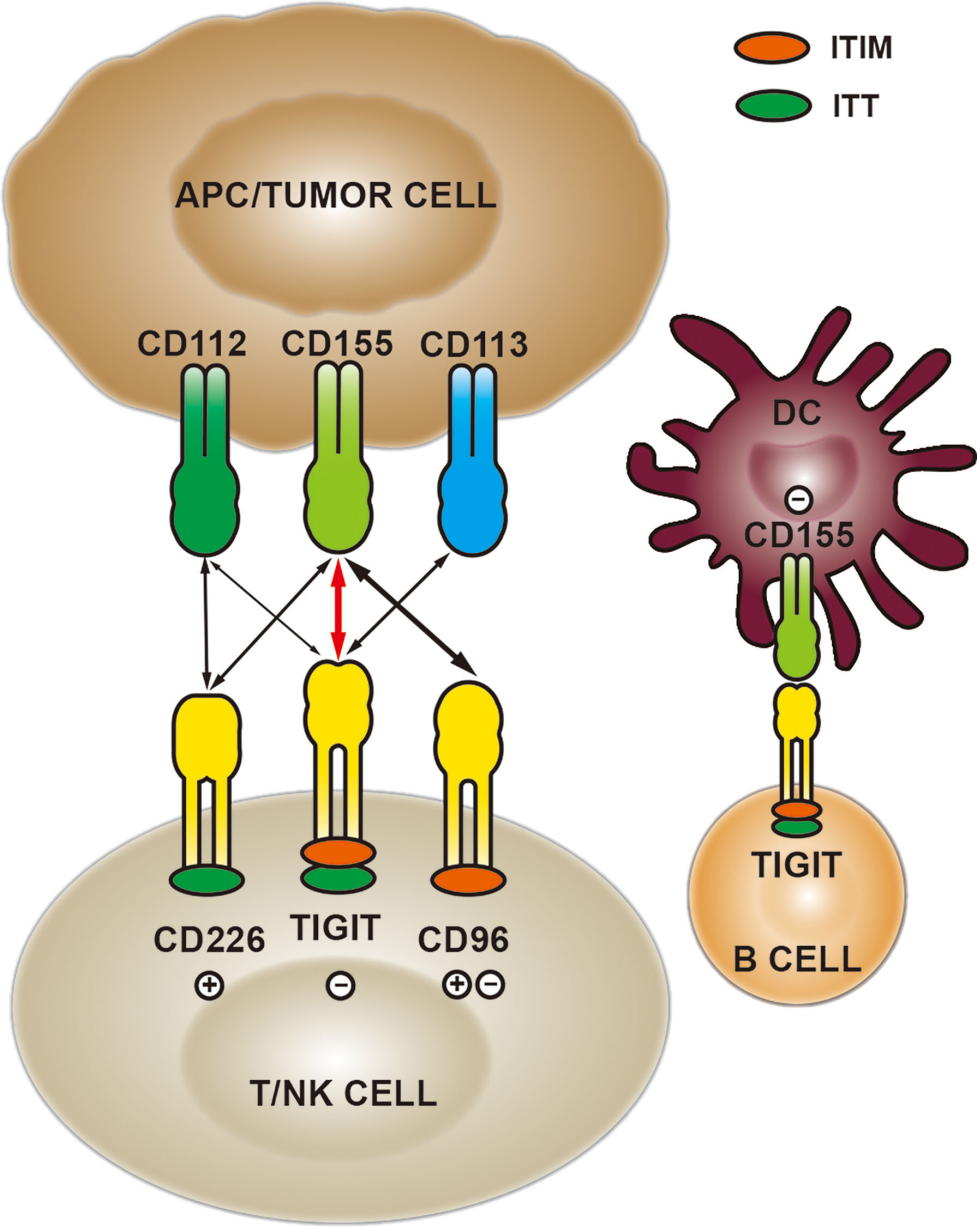
The expression and interaction of TIGIT/CD155 in the tumor microenvironment.

TIGIT is a member of the immunoglobulin superfamily with a molecular structure consisting of extracellular Ig variable region, type I transmembrane domain, intracellular immunoreceptor tyrosine inhibitory motif (ITIM) and immunoglobulin tail tyrosine (ITT)-like motif (25) (**Figure 1**). Current studies reveal that TIGIT is expressed on activated CD8+ T cells, CD4+T cells, regulatory T (Treg) cells and NK cells, and can inhibit the anti-tumor responses mediated by immune cells (26). The latest research has shown that TIGIT is also expressed on B cells, which binds to CD155 expressed on dendritic cells (DC), leading to decreased expression of IL-12, IL-16 and CCR7, thereby inhibiting the maturation and proinflammatory response of DC (27).

CD155, one of the main ligands of TIGIT, exerts functions *via* different mechanisms in tumor and immunology. CD155 is found to be up-regulated in a variety of human malignancies and plays a carcinogenic role by affecting biological functions such as cell adhesion, migration, invasion and proliferation (28). The elevated level of CD155 in human glioma cells modulates the Src/FAK/Paxillin/p130Cas signaling pathway induced by adhesion, promoting cancer cell migration, which is one of the ways for tumor cells to acquire aggressiveness (29). Besides, CD155 deletion can synergize with doxorubicin to induce apoptosis of breast cancer cells and suppress cell growth (30). In immunology, CD155 regulates the functions of immune cells by binding to costimulatory immune receptor CD226 (also named DNAM-1) and inhibitory checkpoint receptors TIGIT and CD96 (28, 31). TIGIT competes with CD226 to bind CD155 and inhibits CD226 signal by disrupting CD226 homodimerization (32). It has been reported that CD155, widely expressed in human and mouse tumor-infiltrating myeloid cells, promotes tumor cell growth and metastasis by down-regulating the expression of CD226 and inhibiting the effects of CD8+T and NK cells (33). Like TIGIT, CD96 is an inhibitory receptor binding to CD155, which can induce NK cell exhaustion and mediate the adhesion function of immune cells (34). Additionally, CD96 and CD226 competitively bind to CD155, impairing the function of NK cells (31).

The immune inhibitory receptors share a common ITIM in their cytoplasmic regions, responsible for transmitting inhibitory signals. TIGIT binds to CD155 with a higher affinity than CD226 and CD96 (20, 28, 35). TIGIT binding to CD155 could suppress the activity and function of lymphocytes. The mechanisms mediated by TIGIT/CD155 signaling axis mainly include the following four aspects: 1) TIGIT interacts with CD155 expressed on DC, inducing IL-10 and suppressing IL-12 production, which directly inhibits DC maturation and indirectly reduces the proliferation and function of T cells (20). 2) They directly exert the inherent inhibitory effect of T cells through the recruitment of SHIP1 and SHP2 phosphatases (36). 3) Direct inhibition of NK cell cytotoxicity and cytokine release (37). 4) The lack of CD226 can enhance the TIGIT/CD155 signal in the inflammatory environment, thereby inhibiting the AKT-mTORC1 pathway, stabilizing Foxp3 and maintaining the immunosuppressive function of Treg cells (38). Currently, CD155/TIGIT has shown potential targeted therapeutic effects in a variety of malignancies, such as high-grade serous ovarian cancer (39) and head and neck squamous cell carcinoma (40). In the sepsis model of tumor-bearing mice, TIGIT was overexpressed on Treg and NK cells, binding to CD155 and mediating immunosuppression. Targeting TIGIT significantly improved the survival of mice and the reduction of T cell apoptosis (41). Based on the studies of CD155/TIGIT in tumor and immunology, it is suggested that TIGIT/CD155 is a promising target.

## Regulation of CD155/TIGIT in Immune Cells

### NK Cells

**Figure 2:**
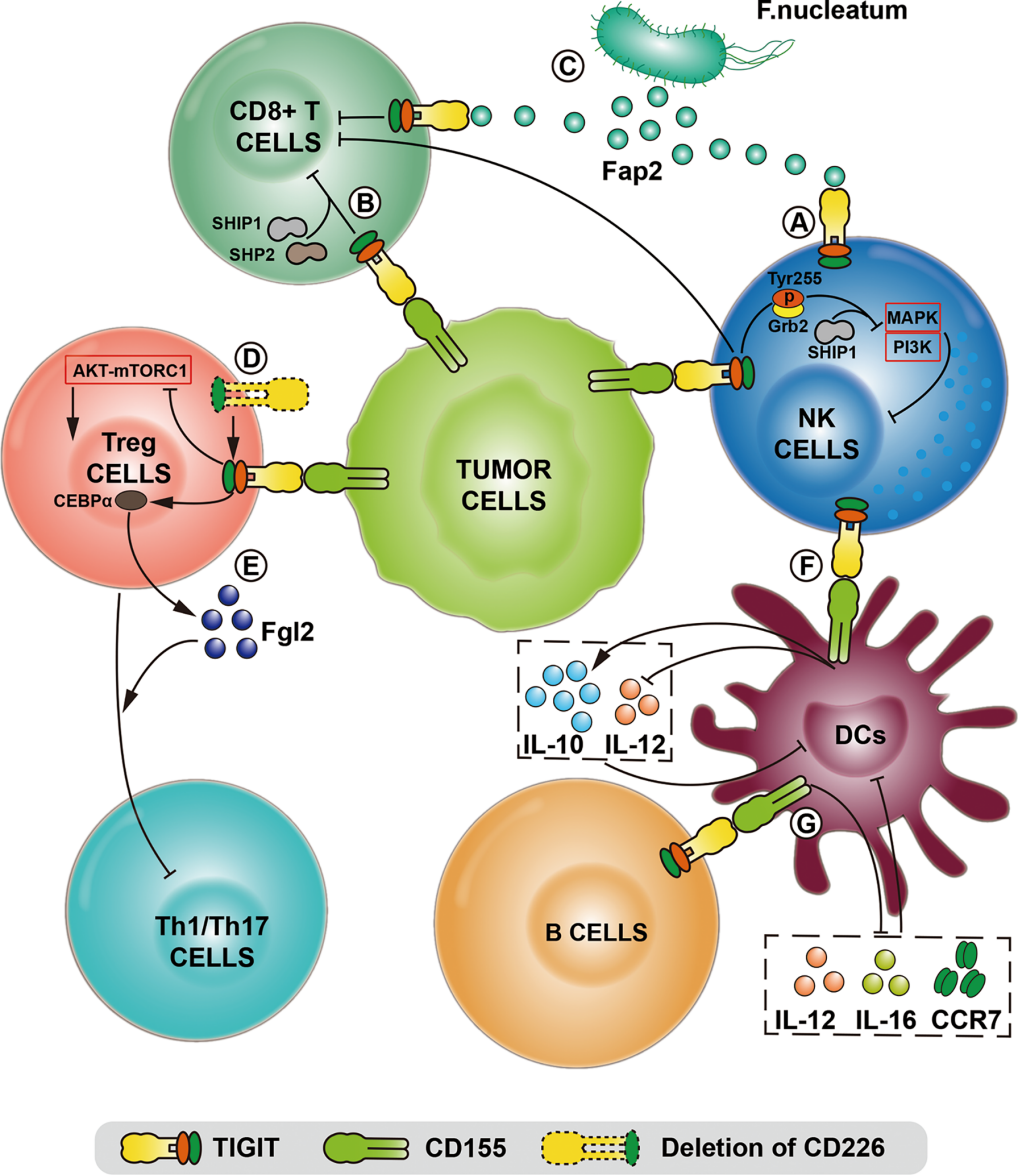
The comprehensive mechanisms of TIGIT/CD155 axis mediated in the immune system in various models and biological contexts. **(A)** After TIGIT expressed on NK cells binds to CD155, the intracellular immunoreceptor (ITT)-like motif of TIGIT is phosphorylated at Tyr255 and interacts with Grb2, which terminates MAPK and PI3K signaling *via* recruiting SHIIP1, leading to the inhibition of NK cell function. **(B)** TIGIT/CD155 axis directly induces CD8+T cell exhaustion by recruiting SHIP1 and SHP2 phosphatase. **(C)** Tumor cells restrict NK and CD8+T cell activity using the Fap2 protein of F.nucleatum bacteria to interact with TIGIT. **(D)** The deletion of CD226 enhances TIGIT/CD155 signaling, thereby maintaining the immunosuppressive function of Tregs with the inhibition of the AKT-mTORC1 pathway. **(E)** After TIGIT/CD155 ligation, the transcription factor CEBPα promotes the transcription of the soluble effector molecule fibrinogen-like protein 2 (Fgl2), thereby the hypersecretion of Fgl2 in Tregs facilitates the suppression of pro-inflammatory Th1 and Th17 cells. **(F)** The binding of CD155 to TIGIT on dendritic cells (DC) induces the production of IL-10 and inhibits the production of IL-12, which directly leads to the DC maturation and indirectly reduces the proliferation and function of T cells. **(G)** TIGIT expressed on specific B cell subsets binds to CD155 expressed on DC, leading to the decreased expression of IL-12, IL-16 and CCR7, thereby inhibiting the maturation and pro-inflammatory response of DC.

NK cells are innate immune cells, which mainly play a role in early infections and inhibiting tumor growth and metastasis. TIGIT, as an inhibitory receptor expressed by all NK cells, recognizes CD155 expressed on cells derived from normal epithelium to protect normal cells from NK cell destruction (37). CD155/TIGIT has been proven to limit the toxic effect and cytokine release function of NK cells *in vitro*. When CD155/TIGIT signal is initiated, the ITT-like motif in the cytoplasmic tail of TIGIT is phosphorylated at Tyr225 and combined with Grb2. Then SHIP1 is recruited to terminate PI3K and MAPK signals prematurely, resulting in the inhibition of NK cell function (**Figure 2**). SHIP1 deletion can significantly reverse the immunosuppression mediated by TIGIT and CD155 (42).

IL-15 signaling plays a critical role in NK survival and differential and can enhance the cytotoxicity mediated by NK cells. One study showed that the combination of IL-15 and anti-TIGIT antibodies improved the immune function of NK cells and T cells (43). Another study confirmed that the combination of IL-15 and TIGIT blockade activated the killing effect of tumor-infiltrating NK cells (44). The blockade of TIGIT in NK cells requires consideration of multiple factors. Previous studies have supported that dual the blockade of PD-1 and TIGIT mainly exerted effects on NK cells, thereby enhancing the anti-tumor activity of CD8+T cells. However, blocking TIGIT alone cannot reverse the cytotoxic effect of NK cells infiltrated in melanoma, while activated NK cells can induce TIGIT expression (31). The anti-tumor effect of blocking TIGIT may also need to consider the activation status of the CD226. CD155 expressed on membrane leads to increased internalization and degradation CD226 on NK cells and promotes NK cell dysfunction. It has been reported that NK cells from malignant ovarian cancer up-regulate TIGIT and down-regulate CD226 expression. Subsequent *in vitro* and *in vivo* experiments have shown that targeting TIGIT can achieve superior efficacy based on up-regulation of CD226 expression (45).

NK cells rely on many receptor combinations to initiate effector function, among which inhibitory receptors recognize MHC-I proteins. Thus, cells expressing MHC-I proteins could be protected from NK cell-mediated lysis. Since CD8+T cells mediate immune destruction of MHC-I presenting tumor cells, NK cells are effective against MHC-I deficient tumors. Simultaneous activation of NK cells and CD8+T cells can be considered an anti-tumor therapy strategy. Therefore, targeting TIGIT/CD155 signaling axis may simultaneously enhance the effector function of CD8+T cells and NK cells, which could be a potential treatment option.

### CD8+T Cells

As the primary anti-tumor effector cells, CD8+ T cells have become a hot spot for immune checkpoint blocking therapy in recent years. In the malignant tumor microenvironment, the infiltrating proportion of CD8+ T cells and the expression pattern of related immune checkpoints affect the prognosis of patients and the response to immunotherapy (46, 47). In mice and humans, including bladder cancer (48), melanoma (49), gastric cancer (50), colorectal cancer (51) and other tumors (52), the expression of TIGIT on infiltrating CD8+ T cells is related to patient survival.

CD155 and TIGIT affect function and survival of CD8+T cells *via* multiple mechanisms. TIGIT can inhibit the NK cell-mediated immune response by binding to CD155, thereby suppressing the anti-tumor effect of CD8+T cells (53). The infiltrating CD8+ T cells in metastatic melanoma have increased expression of TIGIT, which induced the downregulation of costimulatory factor CD226. The corresponding ligand CD155 was elevated in melanoma cells and involved in the malignant progression of the tumor (49).

TIGIT can also mediate tumor immune escape in a bacteria-dependent manner, in which tumor cells directly interact with TIGIT to restrict NK and T cell activity using the Fap2 protein of Fusobacterium nucleatum (54). In a combined analysis of 155 patients with metastatic melanoma treated with ICIs, it was found that poor response to immunotherapy was associated with high expression of CD155 (55). CD155 deletion enhanced tumor sensitivity to PD-1 blockade, which was observed in a vivo non-small cell lung cancer study. It may be attributed to the tumor-infiltrating immune cells, especially the more frequent expression of PD-1 in Tregs. Secondly, CD155 deletion increases the anti-tumor activity of CD8+T cells and NK cells in tumors (56). This feature targets CD155/TIGIT combined with PD-1 blocking therapy to improve the immune response. A chimeric antigen receptor (CAR) T cell targeting CD19 that integrates PD-1 and TIGIT has shown effective anti-tumor activity in relapsed or refractory large B-cell lymphoma, and significantly enhanced CAR-T persistence cells *in vivo* (57).

### Treg Cells

Treg cells are a subset of CD4+T cells characterized by the expression of Foxp3, CD25 and CD4, which play an essential role in regulating immune response and maintaining immune tolerance. Treg cells expressing TIGIT can serve as a subset of cells with activating phenotypic function. TIGIT signal in this cell subset promotes the transcription of the soluble effector molecule fibrinogen-like protein 2 (Fgl2) by inducing the transcription factor CEBPα, thereby the hypersecretion of Fgl2 facilitates the suppression of pro-inflammatory Th1 and Th17 cells mediated by Treg cells (58). Activated Treg cells express TIGIT infiltrating in the tumor microenvironment, which is related to poor survival (59). γδTregs, a new subset of Tregs, has been reported that higher TIGIT+ γδTregs contributed to the poorer overall survival in patients with acute myeloid leukemia, suggesting that TIGIT may be involved in regulating Treg cells in malignancies (60). Besides, highly infiltrated TIGIT+ Treg cells and high levels of CD155 jointly mediate the enhanced inhibitory function of Treg cells in the tumor microenvironment of melanoma (61). The co-stimulatory receptor CD226 competes with TIGIT, and the deletion of CD226 can also enhance Treg cell function mediated by TIGIT (38).

## Role of CD155/TIGIT in Digestive System Cancers

### Gastric Cancer

Gastric cancer is one of the most malignant tumors of the digestive system, and the five-year survival rate remains low (62). The immune microenvironment of gastric cancer is characterized by high heterogeneity and complexity (63), and there is also a high incidence of somatic mutations (64), suggesting that gastric cancer may benefit from immunotherapy. Therefore, identifying novel immune-related therapeutic targets may improve the immune microenvironment and the survival of gastric cancer patients. A study based on clinical samples showed that the elevated expression of TIGIT and CD155 in gastric cancer tissues was closely associated with poor prognosis of patients and functional exhaustion of highly infiltrated CD8+T cells (65). Thus TIGIT/CD155 may be a potential therapeutic target and prognostic marker for gastric cancer.

In human tissues, there is a soluble form of CD155 (sCD155) (66). Membrane binding protein is encoded by CD155α and CD155δ, while sCD155 is encoded by CD155γ and CD155β, and lacks a transmembrane region. However, their significance varies in gastric cancer. The increased expression of sCD155 in the serum of patients with gastric cancer was related to the degree of tumor differentiation (67). sCD155 may serve as a serum marker for the early diagnosis of gastric cancer. It has been reported that sCD155 preferentially binds to CD226 and suppresses CD226-mediated NK cell cytotoxic activity, promoting melanoma metastasis (68). However, it is still unclear whether sCD155 has a similar effect to membrane-expressed CD155 and can combine with TIGIT to generate the immunosuppressive signal.

Chemotherapy is one of the indispensable treatment options for patients with gastric cancer, but it can lead to immunosuppression (69). The high expression of TIGIT on CD8+T cells in patients undergoing chemotherapy is closely associated with the recurrence of gastric cancer, and blocking TIGIT could enhance the proliferation capacity of CD8+T cells and induce the secretion of IFN-γ (70). A recent study demonstrated that the infiltrated CD8+ T cells up-regulated the level of TIGIT in gastric cancer, exhibiting exhausted and decreased metabolic activity. Additionally, these cancer cells suppressed CD8+T cell metabolism *via* CD155/TIGIT signaling, mediated by down-regulation of the AKT/mTOR signaling pathway (50). What needs to be considered is that CD155/TIGIT may mediate cellular immune function in different cell populations including T cells, NK cells and DC cells. TIGIT and PD-1 exerted inhabitation function on CD8+T cell growth, proliferation, and cytokine secretion in gastric cancer patients receiving WT1-targeted DC vaccine. The combination of targeting TIGIT and PD-1 restored DC vaccine-induced activity of T cells (71).

### Liver Cancer

Currently, the FDA approved ICIs for advanced liver cancer include Nivolumab, Durvalumab, and ipilimumab, but only a minority of patients showed a favorable response. Therefore, there is an urgent need to develop new immunotherapies. TIGIT is elevated on lymphocytes infiltrating liver cancer tissue and may participate in the tumor progression (72, 73). Transcriptome analysis of the mouse liver cancer model demonstrated that TIGIT served as a marker of T cell depletion compared with PD-1 (74). Correspondingly, CD155 is up-regulated in liver cancer cells and contributes to the poor prognosis of patients with liver cancer. Besides, high expression of CD155 can induce the expression of TIGIT in CD8+T cells, further suppressing the activation of PI3K, MAPK and NF-κB signaling pathways, thereby reducing the secretion of IFN-γ and TNF-α by CD8+ T cells. Blocking TIGIT can reverse the effector function of CD8+T cells (75). In addition, bacteria are also involved in the interaction of the immune microenvironment of liver cancer. It has been reported that TIGIT and E. multilocularis infection in the liver can induce NK cell depletion and immune escape in tumor microenvironment based on clinical samples and mouse models (76).

Another study indicated that the high expression of CD155 in liver cancer could induce NK cells to up-regulate CD96, leading to impaired NK cell function and inhibition of survival, which is associated with poor clinical prognosis of patients with liver cancer. Blocking the interaction between CD96 and CD155 restored the anti-tumor activities of NK cells (34). Given the recent findings that TIGIT is a significantly expressed immune checkpoint in patients receiving anti-PD-1 treatment, the role of CD155/TIGIT should be noted in the regulation of immune responses. It has been reported that PVRL1 stabilized CD155 expression on the surface of liver cancer cells by reducing the endocytosis of CD155, and then interacted with TIGIT on the surface of CD8+ T cells (77). The anti-PD-1 and TIGIT therapy combination also reversed the proliferation and killing capacities of CD8+ T cells infiltrated by liver cancer, which was observed in another study (73). Based on the treatment of oncolytic viruses targeting PD-1, TIGIT blockade can increase the anti-tumor efficacy of oncolytic viruses (78). The combination therapy may improve the outcome of patients with liver cancer in the future. Therefore, CD155/TIGIT is expected to become a novel target for liver cancer immunotherapy.

At present, therapies targeting CD155/TIGIT are gradually being developed. In a liver cancer ascites model, the construction of the oncolytic virus expressing the soluble extracellular domain of CD155 increased the infiltration of CD8+ T cells and the secretion of IFN-γ, which exhibited the durable tumor-specific immune surveillance. The underlying mechanism was that oncolytic viruses blocked the CD155/TIGIT signal axis and activated CD226 (79). Wang. et al. designed a nanoparticle that blocked the binding of TIGIT and CD155 and targeted LncRNA ANRIL, which exhibited a significant anti-tumor effect and increased the proportion of NK cells and T cells in an *in vivo* model of liver cancer (80). The whole-cell vaccine for liver cancer targeting STAT3 reversed the depletion of T cells and NK cells in the liver cancer microenvironment, which may be attributed to the low expression of PD-1 and TIGIT (81).

Hepatitis B virus (HBV), a risk factors for liver cancer, could induce chronic inflammation, cirrhosis, and ultimately process cancer. The proportion of infiltrated PD-1+ TIGIT+ CD8+ T cells was increased and in a hypo-responsive state in liver cancer patients infected with HBV, especially at advanced stages, which was significantly associated with the poor clinical prognosis (82). This finding indicated that TIGIT might play an essential role in the progression of HBV-related liver cancer. Single-cell sequencing analysis of HBV-related liver cancer showed that tumor-associated macrophages facilitated the formation of an inhibitory immune microenvironment in cancer through the interaction between TIGIT and CD112 (83). Another study confirmed that TIGIT was highly expressed on CD8+ T cells in the HBV immune tolerance mouse model analysis. However, TIGIT blockade or deletion reversed the tolerance of CD8+T cells to viral antigen, which caused chronic hepatitis in mice and eventually led to the development of liver cancer (84). The treatment targeting TIGIT may increase the risk of chronic hepatitis and liver cancer progression. Current studies have shown that antiviral therapy can eliminate the activity of histological necrotic inflammation and improve the prognosis of HBV-associated liver cancer patients (85, 86). We infer that the combination of drugs that improve liver function and antiviral drugs may improve the excessive inflammation caused by the promotion of immunotherapy when immunotherapy targeting CD155/TIGIT is applied to HBV-related liver cancer. Therefore, the protection of liver function and the management of chronic hepatitis should be taken into account in the immunotherapy for liver cancer.

### Pancreatic Cancer

Pancreatic cancer is a highly invasive and malignant tumor. Most patients were non-responders after receiving immune-based treatment strategies, which was attributed to the pathological types of pancreatic cancer were microsatellite stable (87, 88). In recent years, neoantigens have been discovered to be tumor specificity and recognized by the immune system. Previous studies have shown that pancreatic cancer has high-affinity MHC class I-restricted neo-epitopes, and the CD155/TIGIT signal axis is sufficient to facilitate immune evasion mediated by pancreatic cancer expressing neo-epitopes (24). Besides, the infiltrating proportion of T cells is related to the prognosis of pancreatic cancer. Based on single-cell sequencing analysis, the immune microenvironment of pancreatic cancer is characterized by the presence of a large number of immunosuppressive cell populations and functionally exhausted CD8+ T cells that express TIGIT (89). The above findings suggested that the CD155/TIGIT signal axis could serve as the target for pancreatic cancer immunotherapy, especially in patients who do not respond to anti-PD-1 therapy, which is expected to improve the suppressive immune system microenvironment.

A recent study demonstrated that the abundance of CD155 expression in pancreatic cancer tissues was an independent prognostic factor for patients and negatively correlated with the proportion of tumor infiltrating immune cells (90). In the pancreatic cancer microenvironment, NK cells down-regulate the activating receptor molecule CD226 and induce the increased expression of TIGIT (91). Correspondingly, CD155 presents high expression in tumor cells (92). Consistent with this finding, CD8+T cells with elevated expression of CD226 tend to have a more significant response to TIGIT blockade, which indicated that CD226 could be used as a marker for anti-TIGIT therapy (93). Therefore, CD155 may be involved in the progression through immune or non-immune mechanisms, and is expected to become a therapeutic target for pancreatic cancer.

A previous study revealed that the number of NK cells was positively correlated with overall survival of pancreatic cancer patients (94). An *in-vivo* study demonstrated that the increasing infiltration of NK cells in pancreatic cancer could improve prognosis (95). Recently, NK cells and T cells have gained much attention for immunotherapy in pancreatic cancer (96, 97). Current treatment strategies are focused on activating T cells and NK cells after gemcitabine treatment to prevent a recurrence. It has been reported that the application of neoadjuvant administration of PD-1 antagonist combined with gemcitabine improved the viability of NK cells and T cells and inhibited the local recurrence of pancreatic cancer. On this basis, further blocking the immune checkpoint CD96 expressed on NK cells, which bound to CD155, could significantly prevent distant metastasis of pancreatic cancer (98). Based on the above researches, CD155/TIGIT plays an essential role in the unique microenvironment of pancreatic cancer, and targeting this signal axis may become a treatment strategy for pancreatic cancer.

### Colorectal Cancer

Previous studies have demonstrated that TIGIT and CD155 were elevated in colorectal cancer compared with normal tissue (99). The density of CD8+T cells in the tumor margin increased, and expression of PD-1 and TIGIT was up-regulated, compared with that in the tumor center of colorectal cancer (100). Besides, the overexpression of TIGIT in infiltrating lymphocytes exhibited functional failure and hyporesponsive state, which was associated with the advanced stage of tumor (51). High expression of TIGT could lead to impaired glucose metabolism of T cells, and blocking TIGIT restored T cell activity and function (101). Furthermore, the interaction between TIGIT and CD155 participated in regulating CD8+ T cell effector *via* the activation of the NF-κB signaling pathway (102). Thus, TIGIT may serve as an immunotherapy target for colorectal cancer. Interestingly, TIGIT was also expressed on cancer cells of colorectal cancer, but this expression pattern did not affect cell proliferation *in vitro* (103). Given that CD155 is expressed on NK cells and T cells, TIGIT on tumor cells may transmit inhibitory signals to immune cells *via* the interaction with CD155.

Intestinal microorganisms are involved in the symbiotic interaction of the human mucosal immune system. There is a potential connection with the initiation and development of colorectal cancer. Accumulating evidence demonstrated that Fusobacterium nucleatum is enriched in colon cancer and identified as the pro-inflammatory bacteria, contributing to the poor prognosis and low infiltrating T cells in colon cancer (104, 105). A bacteria-dependent immune escape is mediated by cancer cells in the tumor microenvironment. Fusobacterium nucleatum suppressed immune cells cytotoxicity and viability to protect tumor cells from being killed by immune cells through the interaction between Fap2 and TIGIT on NK cells and T cells. The binding site was different from TIGIT and CD155 (54). Fusobacterium nucleatum can also inhibit the activity of T cells and NK cells by binding to the inhibitory receptor CEACAM1 (106). In the future, combinatorial therapy can be considered to treat tumors colonized by Fusobacterium nucleatum.

Furthermore, chronic inflammation mediated by intestinal flora is also a risk factor for colorectal cancer. As a classic non-steroidal anti-inflammatory drug, Aspirin inhibits colorectal cancer cell proliferation and induces tumor cell apoptosis through TIGIT/CD155 signaling axis (107). Previous studies have shown that specific intestinal flora can affect patients’ response to ICIs and contribute to the occurrence of colitis after receiving immune checkpoint treatment (108, 109). In addition, targeting CD155/TIGIT may induce excessive activation of infiltrating CD8+T cells in tumors, leading to excessive inflammation. Therefore, targeted therapies need to consider the effect of intestinal flora or enhance the anti-tumor efficacy by regulating intestinal flora or combining anti-inflammatory drugs.

Colorectal cancer often displays DNA mismatch repair deficiency or microsatellite instability-high, so that tumor cells often express many neoantigens, which are easily recognized by the immune system. Previous studies indicated that immune checkpoint blocking therapy had shown significant efficacy in colorectal cancer patients with DNA mismatch repair deficiency or microsatellite instability-high (110). Immunotherapy shows excellent promise in specific colon cancer genotypes. It has shown that the expression of PD-1 and TIGIT is elevated in colorectal cancers that are defective in mismatch repair (111). When used in combination with PD-1 blockade, blocking TIGT enhances the efficacy of radiotherapy for colorectal cancer (112). CD155 was significantly enriched in colorectal cancer with KRAS mutations, and down-regulated CD155 inhibited the growth of tumor cells (113). However, it is worth noting that 85% of patients with colorectal cancers are mismatch-repair proficient or microsatellite instability-low, displaying no response to immune checkpoint inhibitor therapy (114). Given these, it is necessary to explore new immune checkpoints and deeply investigate the resistance mechanism of immunotherapy in specific genotypes of colon cancer. The efficacy of targeting the TIGIT/CD155 signal axis may depend on further genotyping in colorectal cancer.

### Other Digestive Cancers

Consistent with that in gastric cancer, sCD155 in serum can be used as a biomarker for esophageal cancer. The expression level of sCD155 is closely correlated with the response and prognosis of patients with esophageal cancer after receiving chemotherapy (115). The regulatory mechanism of CD155/TIGIT in esophageal cancer still needs further study. As a malignant tumor with a poor prognosis, the expression of CD155 is elevated in gallbladder cancer, which contributes to the unfavorable prognosis of patients. TIGIT has been observed increased expression in immune cells infiltrated in cancer (116). Thus, CD155/TIGT might become a novel target for immunotherapy of gallbladder cancer. Cholangiocarcinoma and head and neck squamous cell carcinoma, including oral cancer, pharyngeal cancer and laryngeal cancer, exhibit an inhibitory immune microenvironment, in which TIGIT is positively expressed (117, 118), suggesting that targeting TIGIT may be with a promising application prospect.

## Conclusions and Perspectives

The current evidence shows that CD155 and TIGIT are highly expressed in various malignant tumors and can be used as potential biomarkers and therapeutic targets. This signal axis mainly suppresses the survival activity and anti-tumor responses of NK and CD8+ T cells through multiple mechanisms and mediates the immune escape of tumor cells. Recent studies indicate that TIGIT is also expressed on tumor cells and involved in the regulation of B cells, and the role of TIGIT and the underlying mechanism remains to be further investigated. CD155/TIGIT is expected to become a new target for immunotherapy of digestive cancers. The treatment strategy for targeting CD155/TIGIT is still at the preclinical research stage, mainly presented as monoclonal antibody therapy. Considering that emerging immunotherapies include ICIs, DC vaccines, adoptive cell therapy, they can be applied to target CD155/TIGIT in the future to achieve better clinical efficacy. Previous studies have supported the combination of blocking TIGIT and PD-1 can further enhance the response of immunotherapy. Therefore, combining ICIs may be one of the potential strategies for treating malignant digestive cancers. Given the differences between individuals of digestive system tumors, it is necessary to comprehensively consider the effect of tumor genotyping, intestinal flora and inflammation on treatment efficacy in future clinical practice.

## Author Contributions

All authors conceptualized and wrote the manuscript. YL conceived and modified the structure of this review. DW and YG additionally performed literature analysis and figure preparation. All authors contributed to the article and approved the submitted version.

## Funding

This work was supported by the National Natural Science Foundation of China under Grant NO.31770537, the Natural Science Foundation of Gansu Province under Grant NO.20JR10RA740, and the Natural Science Foundation of Gansu Province under Grant NO.20JR5RA345.

## Conflict of Interest

The authors declare that the research was conducted in the absence of any commercial or financial relationships that could be construed as a potential conflict of interest.

## Publisher’s Note

All claims expressed in this article are solely those of the authors and do not necessarily represent those of their affiliated organizations, or those of the publisher, the editors and the reviewers. Any product that may be evaluated in this article, or claim that may be made by its manufacturer, is not guaranteed or endorsed by the publisher.
